# De Novo Assembly and Comparative Mitochondrial Genome Analysis of Three Salt Marsh Specialist Ground Beetle Species (Coleoptera: Carabidae)

**DOI:** 10.1002/ece3.74195

**Published:** 2026-08-20

**Authors:** Anne Mack, Michael Kuhlmann, Christine Ewers

**Affiliations:** ^1^ Zoological Museum, Kiel University Kiel Germany

**Keywords:** Carabidae, de novo assembly, mitochondrial genome

## Abstract

Ground beetles (*Carabidae*) are traditionally used as bioindicators in ecological and evolutionary studies. However, few complete mitochondrial genomes are currently available for this group. In this study, 10 individuals of the salt marsh‐specialist species *Bembidion minimum*, *Dicheirotrichus gustavii*, and *Pogonus chalceus* were sequenced, resulting in nine successfully de novo assembled mitochondrial genomes. The specimens were collected from coastal salt marsh habitats in northern Germany, where they occur in similar coastal environments and represent sympatric salt marsh‐associated Carabidae. Multiple individuals per species were included to ensure assembly consistency and improve robustness of the de novo mitochondrial genome reconstruction. All nine successfully assembled mitochondrial genomes ranged from 16,204 to 17,398 bp in length and contained the typical and identical set of 13 protein‐coding genes, 22 tRNA genes, and 2 rRNA genes, all arranged in the conserved insect gene order. All assembled genomes showed a strong AT‐bias with GC contents of approximately 20%, which was also reflected in the relative synonymous codon usage (RSCU), as preferred codons frequently ended with adenine. Phylogenetic analyses consistently recovered strongly supported monophyletic clades for all three species (BS = 100), while interspecific relationships within Carabidae were weakly to moderately supported, with bootstrap values ranging from approximately 27 to 66. These results indicate that these carabid mitochondrial genomes follow the general structural and compositional patterns known from other Coleoptera. These assemblies add to the limited mitochondrial reference data currently available for Carabidae and may support future comparative, population genetic, and evolutionary analyses.

## Graphical abstract

We assembled and analyzed the complete mitochondrial genomes of 10 individuals from the salt marsh ground beetles *Bembidion minimum*, *Dicheirotrichus gustavii*, and *Pogonus chalceus*, revealing the conserved insect gene order, the typical set of 37 mitochondrial genes, and a pronounced AT‐bias (~80%). Phylogenetic analyses supported monophyletic clades for all three species and expanding the limited mitochondrial reference data available for Carabidae to support future comparative and evolutionary studies.

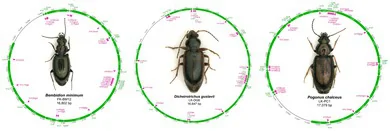

## 1 Introduction

Ground beetles (Carabidae) are one of the most species‐rich beetle families, with over 39,000 species described worldwide (Lorenz 2005). The ecology of this diverse group has been extensively researched, which underpins their traditional use as indicators in ecological and evolutionary studies (Lindroth 1945; Thiele 1977). The species *Bembidion minimum, Pogonus chalceus*, and *Dicheirotrichus gustavii* examined in this study are adapted to salt‐influenced (coastal) habitats (Turin et al. 2022), which represent ecologically challenging and highly selective environments, thereby promoting physiological and evolutionary specialization. *P. chalceus* occurs exclusively on the coasts of the Wadden Sea, while the other species can also be found in salt‐influenced habitats on the Baltic Sea or inland (Tolasch and Gürlich 2026). These salt marsh habitats have been significantly reduced and degraded in many parts of Europe because of coastal development, land reclamation, drainage, and the increasing impacts of climate change (Bakker et al. 2020; Gedan et al. 2009; Vos and Knol 2015). These carabid species are of particular interest as they belong to the group of ground beetles specialized in these specific habitats, which have so far been inadequately studied and for which no complete mitochondrial reference genomes are currently available. Furthermore, they are characteristic species of Atlantic salt marsh ecosystems, and are regarded as typical representatives (Turin et al. 2022) and are considered as indicator species of this habitat type (Desender et al. 1998; Desender and Verdyck 2001; Dhuyvetter et al. 2005) They could therefore be of significance as reference taxa for future biodiversity monitoring and for comparative studies of salt marsh fauna.

The mitochondrial genome is particularly well suited for molecular genetic studies due to its maternally inherited, non‐recombining, and compact structure, as well as its comparatively high mutation rate (Lodish 2021). The length of the circular mitochondrial genome varies in Carabidae between 15 kb (*Notiophilus quadripunctatus*, NC_064369.1) and 25 kb ( *Nebria brevicollis* , OX122912.1) and consists of 37 genes, 13 of which are protein‐coding (PCGs), 2 rRNA genes, and 22 tRNA genes (Cameron 2014). Moreover, there is a non‐coding region of variable length, presumably the origin of transcription initiation (Cameron 2014). The order of the genes is highly conserved in insects and deviations have not yet been documented in carabids (Andújar et al. 2019; Kieran 2020; Lu et al. 2023). Such rearrangements could provide insights into the evolution and phylogeny of the species, as for example in the beetle family Scarabaeidae (Zhang et al. 2025). While the conservation of gene order and structural variations are frequently discussed in a phylogenetic context (e.g., Zhang et al. 2025), the present study focuses on the generation and validation of complete mitochondrial genome assemblies, rather than on testing evolutionary hypotheses or addressing population genetic questions.

Despite their high relevance, relatively few complete mitochondrial genomes of ground beetles are available. Currently, only 237 records of complete and partial mt genomes with more than 8 kb, representing about 200 taxa, are available in the public NCBI database (National Library of Medicine (US) 2025: search conducted in March 2026). Given the enormous diversity of species, with almost 39,000 carabid species occurring worldwide (Lorenz 2005), this covers only 0.5% of the species. This represents a significant gap in the mitochondrial reference data, which limits reliable genomic comparison, the consistency of annotation and molecular identification within the Carabidae.

An expanded genome dataset is not only important for phylogenetic and taxonomic analyses, but also for the optimization of molecular detection methods. Studies show that the established COI standard primers LCO1490/HCO2198 often amplify non‐specifically and detect DNA from symbiotic organisms (Becker et al. 2021). Similarly, COI sometimes exhibits low universal amplification, while other mitochondrial markers such as 12S or 16S provide significantly better results in many insect groups (Marquina et al. 2019). Complete mitochondrial genomes therefore enable the development of more specific primers, improve metabarcoding approaches, and reduce misclassifications in biodiversity‐related studies. They also provide standardized reference resources for future comparative genomic applications in Carabidae.

In this study, the complete mitochondrial genomes of the three salt‐marsh‐associated species *Bembidion minimum, D. gustavii* and *P. chalceus* are sequenced and assembled de novo for the first time, with the aim of generating validated reference genomes. To increase reliability in the generated reference genomes, multiple biological replicates per species were included to ensure reproducible de novo assemblies rather than to address intraspecific or geographic variation. In addition to the de novo assembly, the gene arrangement, base composition and relative synonymous codon usage (RSCU) are analyzed, and a phylogenetic tree is constructed including other carabid species. These analyses are performed to provide basic genomic characterization and quality validation of the assembled mitogenomes.

## 2 Material

**FIGURE 1:**
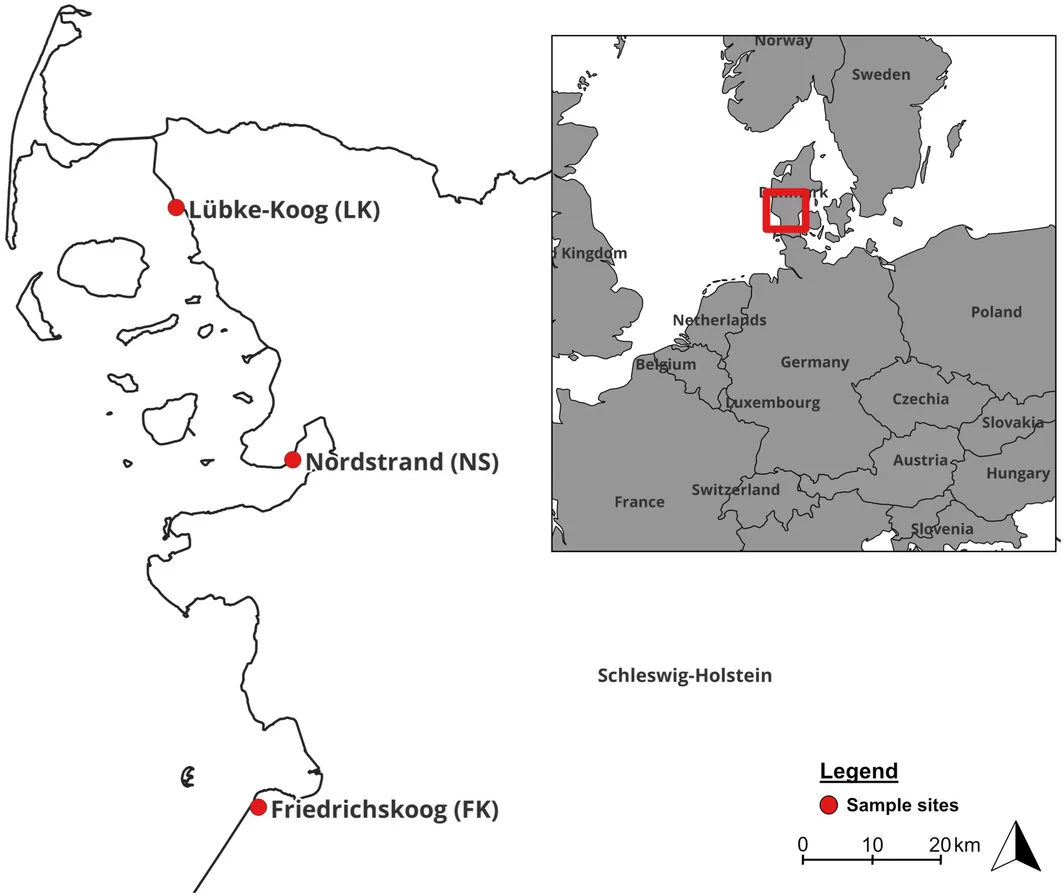
Map of sample sites in northern Germany. Inset map shows the broader geographic affinity of the sampling sites within north‐western Europe with a red box indicating the sampled region. Red dots represent sampling sites.

Carabidae were collected in localities along the Wadden Sea coast in Schleswig‐Holstein in 2024. Figure 1 shows a map of the sampling sites. The map was created with QGis (Version 3.32.2‐Lima, QGIS Association). Detailed collection sites and their coordinates (coordinate system ETRS89/UTM zone 32 N (EPSG:4647(zE‐N))) can be found in Table 1. The sample site “Lübke‐Koog” is an area located in the vicinity and not the polder itself. Permission to collect carabids using pitfall traps was issued by the State Agency for the Environment of Schleswig‐Holstein. Entry permission for all sampling sites was granted by the Schleswig‐Holstein Wadden Sea National Park.

**TABLE 1:** List of sequenced specimens.

Species	Sample site	Sampling period	Sex	Body length [mm]	Sample id
*Bembidion minimum* (fabricius, 1792)	Friedrichskoog (54°01′22.9″N 8°50′50.1″E)	26.07.2024*	M	2.8	FK‐BM12
		25.09.2024*	F	3.1	FK‐BM15
*Dicheirotrichus gustavii* crotch, 1871	Friedrichskoog (54°01′22.9″N 8°50′50.1″E)	11.06.–05.07.2024	M	6.4	FK‐DG2
		26.07.2024*	F	6.9	FK‐DG9
	Lübke‐Koog (54°47′44.1″N 8°39′29.4″E)	25.09.2024*	F	6.7	LK‐DG6
			M	5.9	LK‐DG10
*Pogonus chalceus* (marsham, 1802)	Lübke‐Koog (54°47′44.1″N 8°39′29.4″E)	25.07.2024*	M	5.9	LK‐PC1
			M	5.6	LK‐PC2
	Nordstrand (54°28′07.9″N 8°55′20.1″E)	22.05.–11.06.2024	F	5.8	NS‐PC1
			M	5.8	NS‐PC2

*Note:* Sampling periods with (*) indicate hand collection. Others are sampled with pitfall traps with propylene glycol as trapping liquid. Sex: F = Female. M = Male. Body lengths were measured from the posterior margin of the labrum to the posterior end of the elytra.

Carabidae were collected either with pitfall traps (9 cm diameter) that were half‐filled with propylene glycol (50%) as a trapping liquid that were emptied every three weeks or by hand collection with an aspirator. The specimens were transferred to a screw‐top jar with 75% ethanol until they were processed in the laboratory.

## 3 Methods

### 3.1 Species Identification

In the laboratory, specimens were examined with the stereomicroscope Zeiss Stemi 508 (Carl Zeiss AG, Oberkochen, Germany). All Carabidae were identified according to Freude (2004), and the nomenclature follows that work. Body lengths were measured from the posterior margin of the labrum to the posterior end of the elytra under a stereomicroscope with a measuring eyepiece. Samples were placed in 75% ethanol until extraction. Photographs of the sampled species were taken with the digital microscope Keyence VHX‐5000 (KEYENCE DEUTSCHLAND GmbH, Neu‐Isenburg, Germany). For editing the images, the software GIMP 2.10.38 (GNU Image Manipulation Program, Copyright 1995–2024) was used.

### 3.2 DNA Extraction and Whole Genome Sequencing

DNA was extracted from whole specimens with the DNeasy Blood and Tissue Kit (QIAGEN GmbH, Hilden, Germany, Cat. No. 69506). Samples were briefly homogenized with a micro pestle (Carl Roth, Karlsruhe, Germany, Art. No. CXH9) before digestion. The incubation step with proteinase K was performed overnight. In addition to the manufacturer's standard protocol, a washing step with 80% ethanol, dry spin and incubation for 10 min was performed to dry the membrane of the spin column before elution. A total of ten samples were submitted to the Competence Centre for Genomic Analysis Kiel (CCGA) for whole genome sequencing (see Table 1). Male and female specimens were included solely as available biological replicates. Sex was not considered an experimental factor, and no sex‐specific analyses were performed. Multiple biological replicates per species were included to support the generation of reliable de novo assemblies and to reduce the likelihood that assembly artifacts or technical variation would influence the resulting mitochondrial reference genomes. The sampling design was not intended to investigate population genetic structure, geographic differentiation or sex‐specific differences. Sequencing was performed on one lane of the Illumina NextSeq1000 sequencer with 300 cycles.

### 3.3 De Novo Assembly of Mitochondrial Genomes

Raw data from whole‐genome sequencing (WGS) were first cleaned. This involved removing sequence adapters and bases with a Phred quality score below 20 using the AdapterRemoval programme (Lindgreen 2012). Baited de novo assembly was performed with NOVOPlasty (Dierckxsens et al. 2017; Version 4.3.5) using publicly available species‐specific COI sequences from ncbi database as seeds: *D. gustavii* (KU916612.1), *P. chalceus* (KJ371157.1) and *B. minimum* (HQ164713.1). Since the de novo assembly for FK‐DG9 with NOVOPlasty was not successful, it was completed by a supplementary assembly with GetOrganelle (Jin et al. 2020; Version, 1.7.7.1). The mitochondrial genomes were annotated using MITOS2 (Bernt et al. 2013). With this tool, the tRNA gene for the amino acid phenylalanine was missing from all assemblies of *D. gustavii* which was supplemented using ARWEN due to the higher sensitivity in detecting mitochondrial tRNA genes (Laslett and Canbäck 2008). Protein‐coding genes (PCGs) and rRNA genes were checked with BLASTn function of NCBI database (Benson et al. 2005). Furthermore, nucleotide sequences were translated into protein sequences and manually checked for internal stop codons and extended to the next stop codon in Geneious Prime (Version 2025.2.2) (Kearse et al. 2012). Illustration of mitochondrial genomes were made with Geneious Prime as well as the calculation of the overall GC contents of all PCGs. For genetic species identification, a BLASTn search of COX1 genes of the newly assembled mitochondrial genomes was performed using the NCBI online tool with default settings (Altschul et al. 1997). Base skew values of the whole genomes were calculated with the formulas: GC skew = (G − C)/(G + C) and AT skew = (A − T)/(A + T) (Perna and Kocher 1995). To investigate whether differences in mitochondrial genome length were associated with tandem repeats within the control region, analyses of tandem repeats of the control regions were performed with Tandem Repeat Finder (TRF) (Benson 1999) with basic option. Furthermore, the relative synonymous codon usage (RSCU) was computed for 13 protein‐coding genes in R using the package seqinr (Charif and Lobry 2007). A phylogenetic dataset was constructed including the 16 reference mitochondrial genomes retrieved from the NCBI database (see Table S1 in Appendix S1 for accession numbers) and the newly generated mitochondrial genomes. Reference taxa were selected based on the availability of complete mitochondrial genome sequences, reliable taxonomic identification, and adequate representation of major Carabidae lineages. The analysis was rooted using *Dytiscus marginalis* (PQ474423.1) as outgroup. All mitochondrial genomes were aligned as complete nucleotide sequences in Geneious Prime using a global alignment with free end gaps and a cost matrix corresponding to 65% similarity (5.0/−4.0). No partitioning scheme or gene‐based concatenation was applied, intergenic regions were included and no regions were excluded in the alignment. The final alignment comprised 19,761 bp. Phylogenetic reconstruction was performed using the maximum likelihood method implemented in the PHYML plugin in Geneious Prime under the GTR substitution model with nucleotide data with 1000 bootstrap replicates.

## 4 Results

Of the 10 sequenced samples, de novo assemblies of the mitochondrial genome could be calculated for 9 samples (Table 2). The assembly for sample NS‐PC2 of the species *Pogonus chalceus* was unsuccessful. In this case, the longest contig that could be calculated (11,568 bp) by the assembler is provided.

**TABLE 2:** Summary of sequencing and de novo assembly data.

Species	Sample id	Raw reads	Assembler	Aligned reads	Assembled reads	Length mt genome [bp]	Ø Coverage (X)	GC %	AT‐skew	GC‐skew	Accession number in GenBank
*Bembidion minimum*	FK‐BM12	16,468,578	Novoplasty	30,812	24,988	16,802	277	21.2	0.0401	−0.1583	PZ095926
	FK‐BM15	18,759,102	Novoplasty	119,536	101,338	16,806	1074	21.2	0.0405	−0.1575	PZ095927
*Dicheirotrichus gustavii*	FK‐DG2	11,975,476	Novoplasty	3940	3344	17,398	34	21.4	0.0371	−0.1702	PZ095925
	FK‐DG9	17,757,240	GetOrganelle	—	—	16,272	25.3	21.4	0.0274	−0.1565	PZ095929
	LK‐DG6	18,029,516	Novoplasty	44,348	38,326	16,647	402	21.4	0.0303	−0.1590	PZ095928
	LK‐DG10	27,074,362	Novoplasty	26,130	17,832	16,270	242	21.4	0.0270	−0.1549	PZ095933
*Pogonus chalceus*	LK‐PC1	22,713,072	Novoplasty	48,270	38,410	17,079	427	20.7	0.0517	−0.1491	PZ095930
	LK‐PC2	20,306,078	Novoplasty	55,660	46,176	17,079	492	20.7	0.0521	−0.1499	PZ095931
	NS‐PC1	18,690,014	Novoplasty	55,420	45,540	16,204	516	20.7	0.0504	−0.1444	PZ095932
	NS‐PC2	28,579,858	Novoplasty	23,154	11,354	11,568^a^	—	—	—	—	—

^a^ Largest contig.

All individuals showed 13 PCGs, 2 rRNA genes, 22 tRNA genes and a control region of various length, but in same order. The length of the mitochondrial genome of *Bembidion minimum* was around 16,800 bp with coverages of 277× and over 1000× (Table 2). In *Dicheirotrichus gustavii*, the length ranges between 16,270 bp and 17,398 bp with average coverages of over 400× in sample LK‐DG6 to 25.3× in FK‐DG9 (Table 2). *Pogonus chalceus* shows complete mitochondrial genome lengths between 16,204 bp and 17,079 bp, and the data provided a coverage of 400‐500× (Table 2). The GC content of the genomes was slightly above 20% in all samples and remained consistent within each species. It ranged from 20.7% in *P. chalceus* to 21.4% to 21.2% in *B. minimum* to the highest values of 21.4% in *D. gustavii*. AT and GC skew values were highly consistent within each species (Table 2). *B. minimum* showed AT skew values of approximately 0.04, *D*. *gustavii* exhibited slightly lower values (0.0270–0.0371), whereas *P. chalceus* showed the highest AT skew values (0.0504–0.0521). GC skew values were negative and between −0.144 to −0.17 in all samples and varied only slightly among species. Analysis of the tandem repeats in the control regions revealed species‐specific and individual variations. While only short tandem repeats were found in *B. minimum* which were highly similar in both samples, the other species showed more differences. In *D. gustavii*, four repeats of an approximately 376‐bp fragment were found in sample FK‐DG2 and two in LK‐DG6. No comparable repeats were detected in the other two samples of this species. In *P. chalceus* , only shorter repeat regions were identified. The control region of NS‐PC1, which had a shorter mitochondrial genome (approximately 800 bp shorter than LK‐PC1 and LK‐PC2), contained repeat regions that were similar to those in the other samples but differed in arrangement and position. Detailed results on the tandem repeats are listed in the Appendix S1. In the BLASTn search of the COX1 genes from the newly assembled genomes, all samples showed a high degree of similarity (Per. ident ≥ 99.6) to references for the respective species.

**FIGURE 2:**
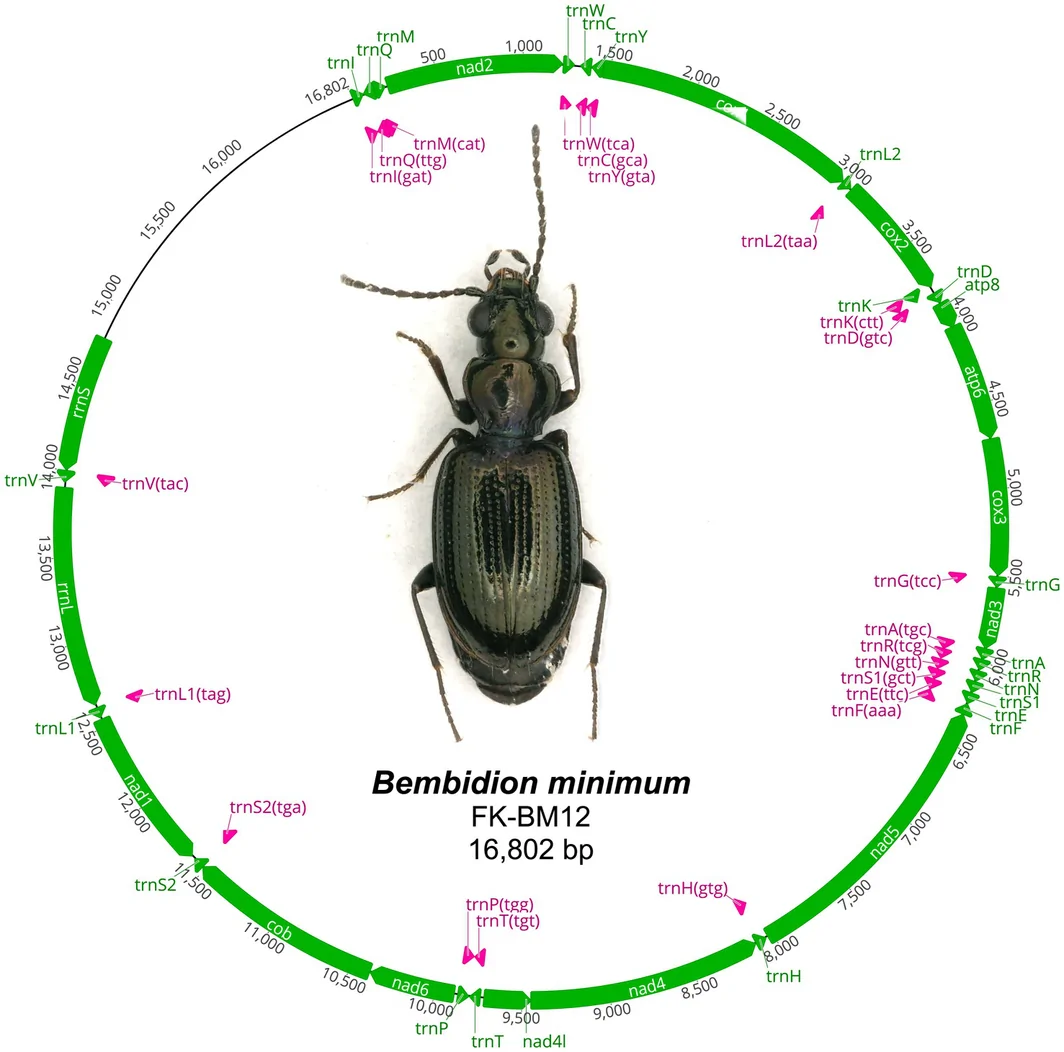
De novo assembled mitochondrial genome of *Bembidion minimum*. Exemplarily one mitochondrial genome for this species is shown here. Sample id = FK‐BM12. Green = Protein‐coding genes. Orange = rRNA genes. Blue = tRNA genes.

**FIGURE 3:**
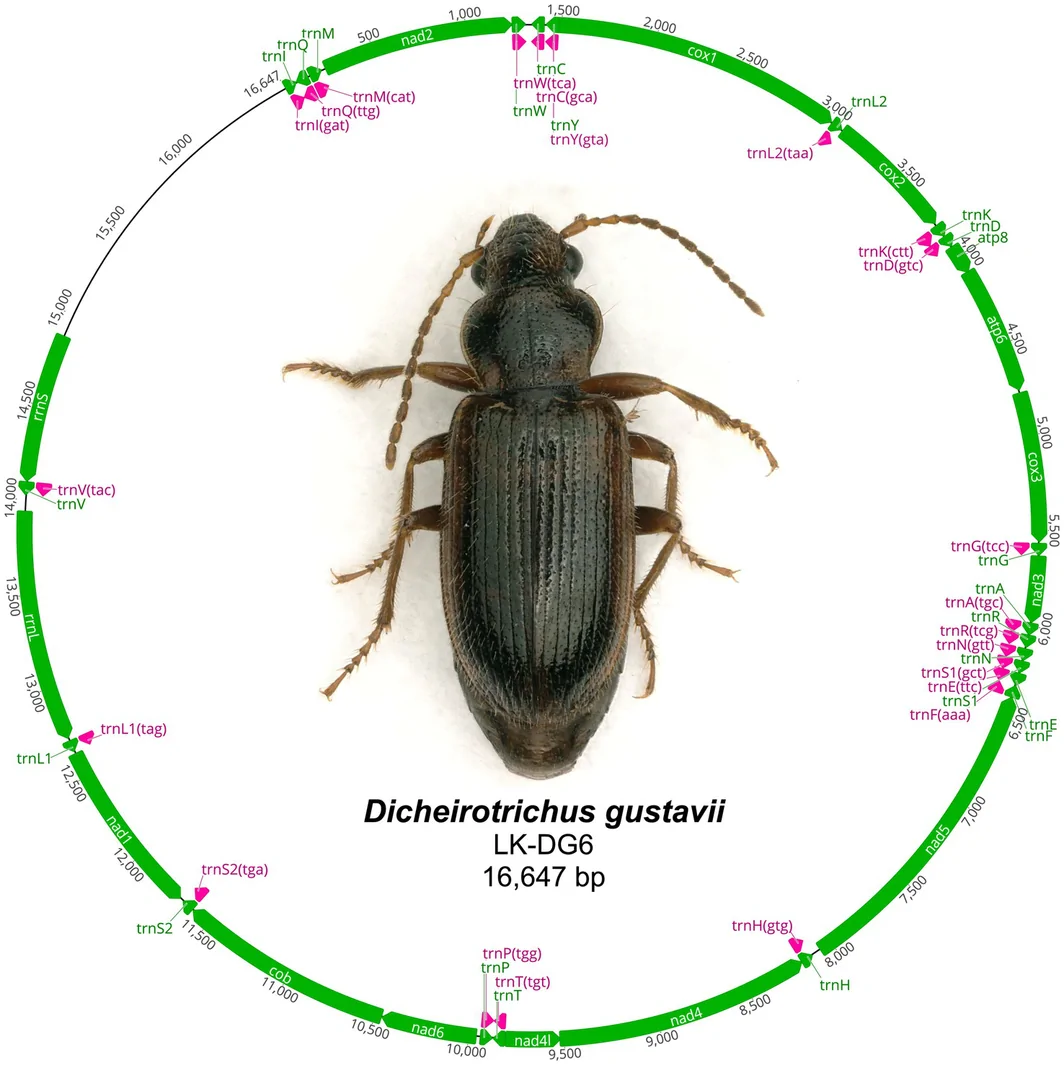
De novo assembled mitochondrial genome of *Dicheirotrichus gustavii*. Exemplarily one mitochondrial genome for this species is shown here. Sample id = LK‐DG6. Green = Protein‐coding genes. Orange = rRNA genes. Blue = tRNA genes.

**FIGURE 4:**
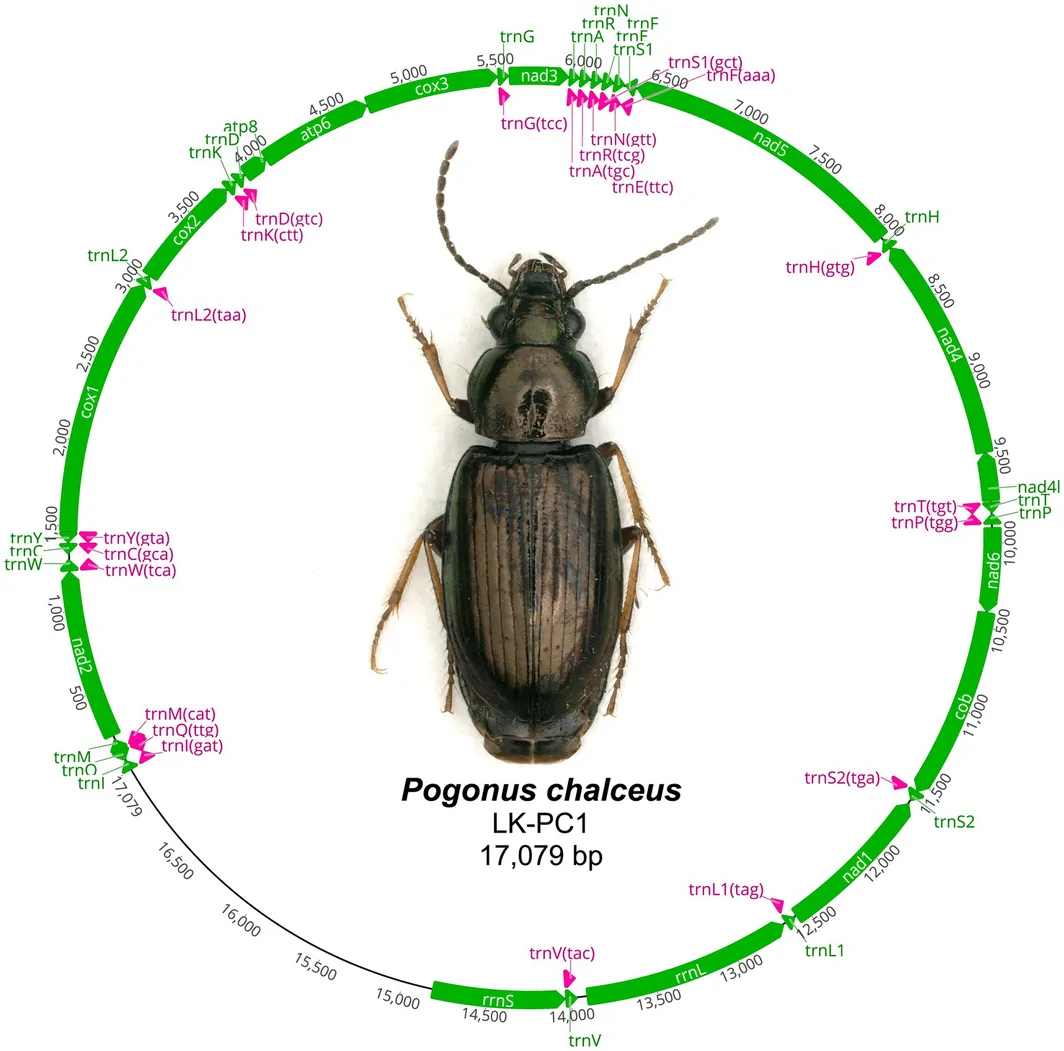
De novo assembled mitochondrial genome of *Pogonus chalceus*. Exemplarily one mitochondrial genome for this species is shown here. Sample id = LK‐PC1. Green = Protein‐coding genes. Orange = rRNA genes. Blue = tRNA genes.

**FIGURE 5:**
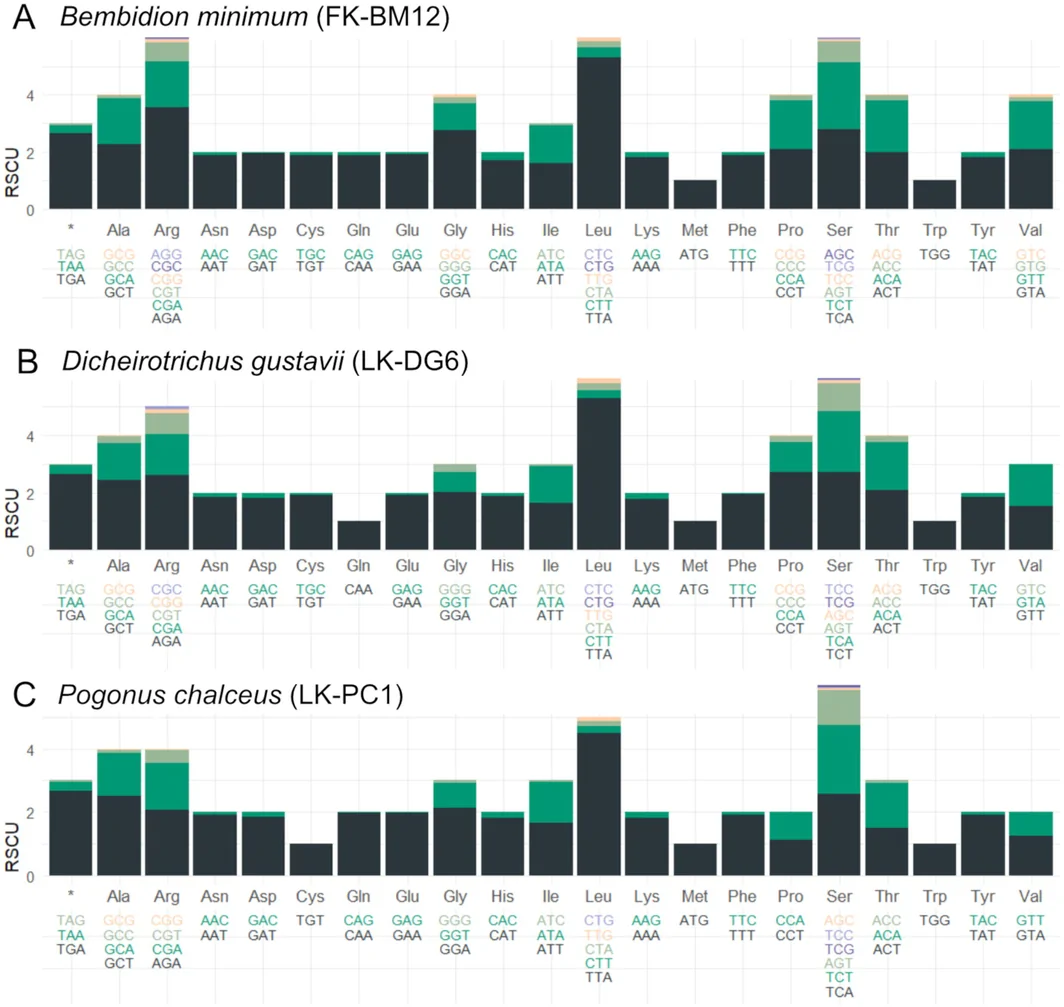
The relative synonymous codon usage (RSCU) for 13 protein‐coding genes (PCGs) for one sample per species. (A) *Bembidion minimum*, sample FK‐BM12. (B) *Dicheirotrichus gustavii*, sample LK‐DG6. (C) *Pogonus chalceus*, sample LK‐PC1. Different colors of the bars indicate the codons used for the respective amino acid.

As the de novo‐assembled mitochondrial genomes are comparable within each species, one is shown in Figures 2, 3, 4, 5 as an example for each of the three species (Figure 2: *Bembidion minimum*, FK‐BM12; Figure 3: *Dicheirotrichus gustavii*, LK‐DG6; Figure 4: *Pogonus chalceus*, LK‐PC1). All de novo assemblies can be found in the Appendix S1.

The RSCU was calculated for 13 protein‐coding genes (PCGs). Since the RSCU values were similar within the species, a representative sample for each species is shown here: *B. minimum* (FK‐BM12, Figure 5A), *D. gustavii* (LK‐DG6, Figure 5B) and *P. chalceus* (LK‐PC1, Figure 5C). RSCU graphs of COI and NAD5 of the other samples are shown in the Appendix S1. All three species showed similar codon usage patterns. Frequently used codons (RSCU > 2) occur particularly in amino acids such as leucine, serine, alanine and arginine and preferentially end in A or T.

**FIGURE 6:**
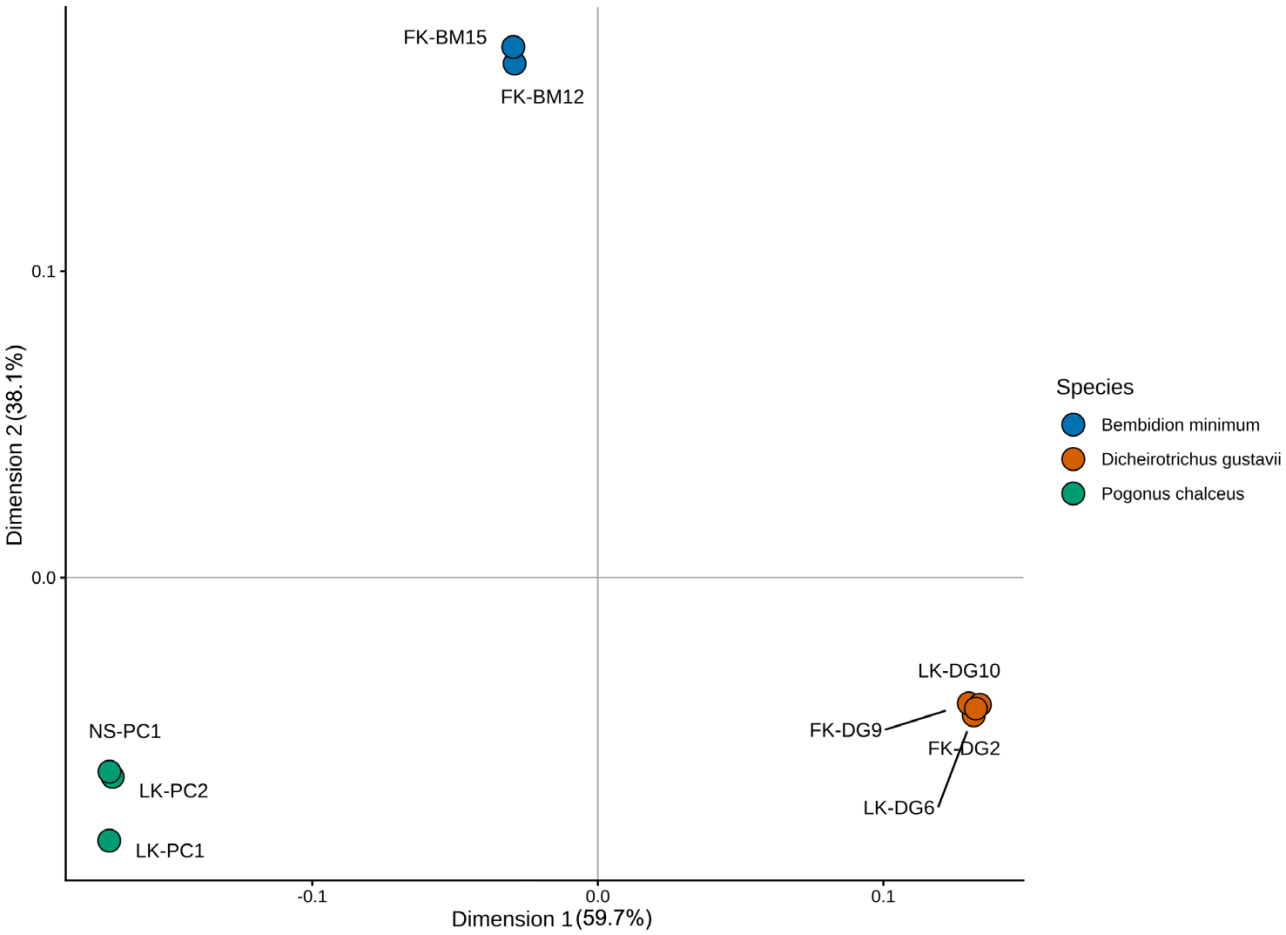
Correspondence analysis (CoA) based on relative synonymous codon usage (RSCU) values calculated from all 13 mitochondrial protein‐coding genes (PCGs). Each point represents one mitochondrial genome, colored according to species. The first two dimensions explain 97.8% of the total variation in codon usage (Dimension 1: 59.7%; Dimension 2: 38.1%).

Furthermore, a correspondence analysis (CoA) based on RSCU values of all 13 mitochondrial protein‐coding genes was performed (Figure 6). The analysis shows three distinct clusters corresponding to the different species, and specimens of the same species are clustered closely together. The first two dimensions explained 97.8% of the total variation.

**FIGURE 7:**
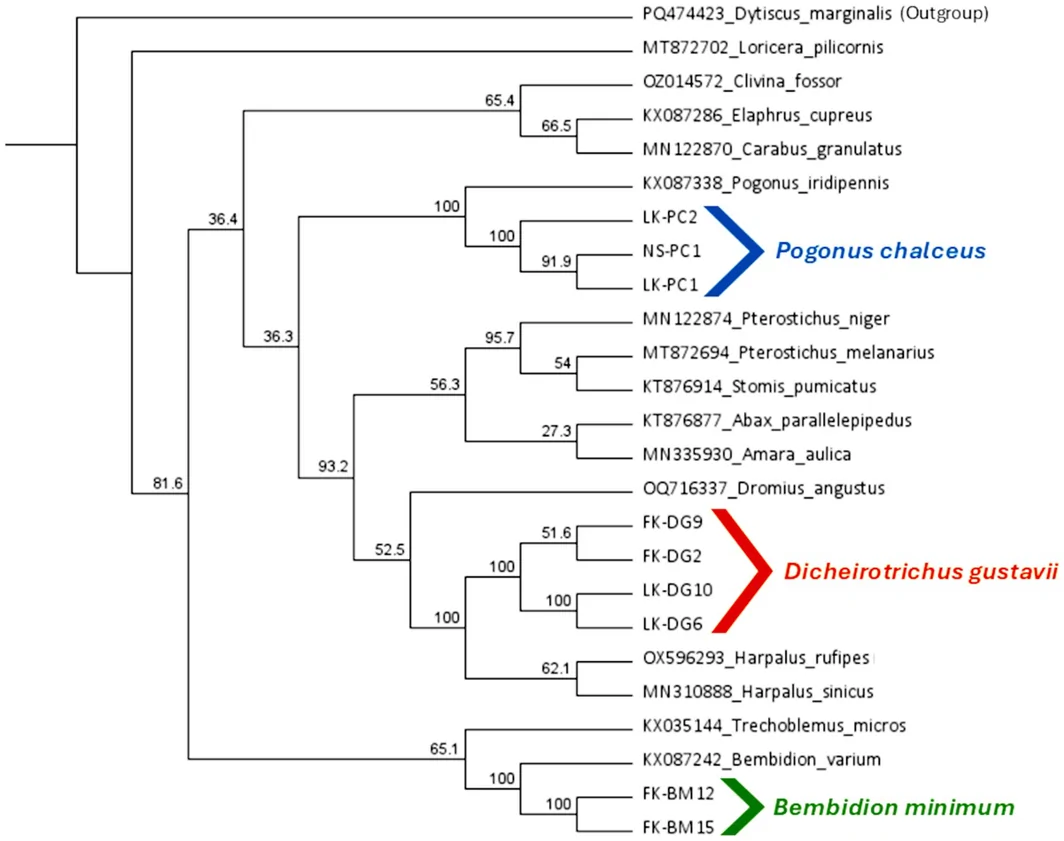
Phylogenetic tree of complete mitochondrial genomes for the Carabidae. Tree was reconstructed with a maximum likelihood approach. *Dytiscus marginalis* serves as outgroup. Numbers on nodes indicate bootstrap support (BS) values based on 1000 replicates.

The phylogenetic analysis is shown in Figure 7. All analyzed specimens of each species formed well‐supported monophyletic clades with high bootstrap support (Figure 7). No clustering according to sex was observed, as females and males clustered together within the respective species clades. Similarly, specimens from different sampling localities were grouped within their respective species. While *D. gustavii* shows a grouping by sampling site (Friedrichskoog and Lübke‐Koog), this is not observed in *P. chalceus* .

Within the phylogenetic tree, all *Pogonus chalceus* samples cluster with *P. iridipennis* while all *D. gustavii* samples group together and appear as sister group to *Harpalus*. The analyzed specimens of *Bembidion minimum* cluster together with *B. varium* .

## 5 Discussion

The de novo assembled mitochondrial genomes show high similarity to previously published insect and beetle mitogenomes. The total A + T content of the mitochondrial genome (78.6%–79.3%) is consistent with values reported for other ground beetles, including *Harpalus pensylvanicus* (80.4%) (Kieran 2020) and *Carabus lafossei* (79.7%) (Chen et al. 2018). In agreement with earlier studies, the A + T bias was particularly pronounced at the third codon position (Yang et al. 2018; Ding et al. 2023; Zhang et al. 2025). This compositional bias leads to a preference for codons ending in A or U, such as UUA (Leu), AUU (Ile) or UUU (Phe), which was also reflected in the relative synonymous codon usage (RSCU) of the analyzed genomes (Chen et al. 2020; Lu et al. 2023; Zhang et al. 2025). The observed values for AT (0.027–0.052) and GC (−0.170 to −0.144) skew were consistent within species and largely constant within taxa, as described previously for Carabidae, e.g., for *Harpalus pensylvanicus* (AT skew = 0.03, GC skew = −0.18) and *Carabus lafossei* (AT skew = 0.033, GC skew = −0.17). Gene order was identical to that reported for other sequenced Carabidae, including *Harpalus pensylvanicus* (Kieran 2020) and *Carabus lafossei* (Liu et al. 2018), with no evidence for gene rearrangements.

The length of the mitochondrial genomes differed mainly due to taxon‐specific variation in the copy number of tandem repeats within the control region (Morgan et al. 2022). In the present study, tandem repeats only partly explained the observed genome length variation within taxa. For example, the shorter mitochondrial genome of *P. chalceus* sample (NS‐PC1) could not be attributed exclusively to differences in the copy number of tandem repeats. This may be attributed to the technical challenges of assembling repetitive regions from short‐read sequencing data, which are known to be difficult regardless of the assembly software used (Dierckxsens, Mardulyn and Smits, 2017). Future long‐read sequencing may circumvent this issue. The included multiple biological replicates per species were therefore useful, as they enabled independent validation of the assembled mitochondrial genomes and increased the robustness of the generated reference sequences. This approach was particularly useful as one sample ( *P. chalceus* , NS‐PC2) could not be successfully assembled. Nevertheless, complete mitochondrial reference genomes were obtained for all three species. This shows that biological replicates can compensate for technical errors in individual samples and thus ensure a successful generation of reference genomes. Furthermore, including multiple replicates also helps to design more robust primers. This enables the identification of potential polymorphisms in the regions where the primers bind, which can then be considered during primer design.

Codon usage patterns were species‐specific and remained largely constant within a species. This was further supported by correspondence analysis (CoA), which was based on the RSCU values of all 13 mitochondrial protein‐coding genes and yielded three clearly distinct clusters corresponding to the species studied. Specimens of the same species clustered closely together, and the first two dimensions explained 97.8% of the total variation, suggesting a high degree of consistency in codon usage patterns between biological replicates.

Phylogenetic analyses consistently recovered strongly supported monophyletic clades for all three species (BS = 100), which demonstrates that independently assembled mitochondrial genomes from multiple individuals resulted in consistent species‐level clustering. No separation according to sex was observed, as male and female specimens clustered together within their respective species clades. The interspecific relationships within Carabidae were weakly to moderately supported, with bootstrap values ranging from approximately 27 to 66, suggesting that the present dataset provides limited resolution of broader carabid relationships. Although all the species studied are associated with saltmarsh habitats, they belong to different lineages within the Carabidae and do not form a distinct evolutionary group. This pattern suggests that specialization in salt‐associated habitats has evolved independently within different evolutionary lineages of Carabidae, highlighting that adaptation to saline environments has occurred multiple times within the family. As the phylogenetic analysis was intended to assess the consistency and taxonomic classification of the newly generated mitochondrial genomes, rather than to elucidate comprehensive evolutionary relationships within the Carabidae, these results support the reliability of the generated reference sequences. The high sequence similarity of the COX1 genes with the corresponding reference sequences in the BLASTn analyses (≥ 99.6%) further supports the morphological species identification and confirms the taxonomic classification of the newly sequenced mitochondrial genomes.

Beyond their application in species identification and phylogenetic placement, the generated reference genomes provide valuable resources for future research on salt marsh‐associated Carabidae. They serve as the basis for future comparative studies, including investigations of evolutionary history, habitat specialization and lineage‐specific responses to saline environments. By expanding the currently limited genomic resources for habitat‐specialized Carabidae, these reference genomes will facilitate future phylogenetic, taxonomic, and molecular ecological studies. Furthermore, they can support biodiversity monitoring, phylogeographic analyses, and conservation genetics approaches for coastal salt marsh ecosystems, which is of particular importance given the ongoing global decline of these highly vulnerable habitats (Duarte et al. 2008).

## Author Contributions

**Anne Mack:** conceptualization (lead), data curation (lead), investigation (lead), methodology (lead), project administration (lead), visualization (lead), writing – original draft (lead). **Michael Kuhlmann:** funding acquisition (lead), resources (equal), writing – review and editing (equal). **Christine Ewers:** methodology (supporting), resources (equal), writing – original draft (supporting), writing – review and editing (equal).

## Conflicts of Interest

The authors declare no conflicts of interest.

## Supporting information


**Appendix S1:** De novo assemblies of mitochondrial genomes.
**Appendix S2:** RSCU graphs for all samples.
**Appendix S3:** List of sequences included in the phylogenetic tree.
**Appendix S4:** Detailed results of analysis of tandem repeats in the control regions.

## Data Availability

All sequencing data except NS‐LK2 is available on NCBI databank (Accession No. PZ095925‐PZ095933). NS‐LK2 is available on request from the authors. All data generated or analyzed during this study are included in this published article [and its Supporting Information files].The arthropod specimens analyzed are deposited at the Zoological Museum Kiel.
