# Supplementary material for: De Novo Assembly and Comparative Mitochondrial Genome Analysis of Three Salt Marsh Specialist Ground Beetle Species (Coleoptera: Carabidae)
Source: Ecol Evol. 2026 Aug 20;16(8):e74195. doi: 10.1002/ece3.74195 (PMC13491389; doi:10.1002/ece3.74195)
Supplement: Supplementary file 1 [file ECE3-16-e74195-s001.docx]

**Supplementary material**

**Appendix S1. De novo assemblies of mitochondrial genomes**

**Figure 1. Sample FK-BM15. Bembidion minimum. Assembled with Novoplasty.**

**Figure 2. Sample FK-DG2. Dicheirotrichus gustavii. Assembled with Novoplasty.**

**Figure 3.** **Sample FK-DG9. Dicheirotrichus gustavii. Assembled with GetOrganelle.**

**Figure 4.** **Sample LK-DG10. Dicheirotrichus gustavii. Assembled with Novoplasty.**

**Figure 5. Sample LK-PC2. Pogonus chalceus. Assembled with Novoplasty.**

**Figure 6.** **Sample NS-PC1. Pogonus chalceus. Assembled with Novoplasty.**

**Appendix S2. RSCU graphs for all samples**

**Figure 7. RSCU for 13 protein-coding genes.** A: Sample FK-BM12. B: Sample FK-DG2. C: Sample FK-DG9. D: Sample LK-DG10. E: Sample LK-PC2. F: Sample NS-PC1. Different colors of the bars indicate the codons used for the respective amino acid.

**Appendix S3. List of sequences included in the phylogenetic tree**

All sequences comprise whole mitochondrial genomes of the respective species. All sequences were downloaded from NCBI database on August 12 2025 (National Library of Medicine (US), 2025).

PQ474423.1 *Dytiscus marginalis* (outgroup)

MT872702.1 *Loricera pilicornis*

OZ014572.1 *Clivina fossor*

KX087286.1 *Elaphrus cupreus*

MN122870.1 *Carabus granulatus*

KX087338.1 *Pogonus iridipennis*

MN122874.1 *Pterostichus niger*

MT872694.1 *Pterostichus melanarius*

KT876914.1 *Stomis pumicatus*

KT876877.1 *Abax parallelepipedus*

MN335930.1 *Amara aulica*

OQ716337.1 *Dromius angustatus*

OX596293.1 *Harpalus rufipes*

MN310888.1 *Harpalus sinicus*

KX035144.1 *Trechoblemus micros*

KX087242.1 *Bembidion varium*

**Appendix S4. Detailed results of analysis of tandem repeats in the control regions**

Table 1. Detailed values of analysis of tandem repeats in the control regions of 9 samples with successful de novo assembly of the mitochondrial genome. Analysis was performed with Tandem Repeat Finder (TRF) (Benson, 1999).

| **Sam-ple** | **Species** | **Indices** | **Per-iod size** | **Copy no.** | **Consen-**  **sus Size** | **% Mat-**  **ches** | **Percent Indels** | **Score** | **A** | **C** | **G** | **T** | **Entro-**  **py**  **(0-2)** |
| --- | --- | --- | --- | --- | --- | --- | --- | --- | --- | --- | --- | --- | --- |
| FK-DG2 | *D. gustavii* | 15041-16492 | 376 | 3.9 | 375 | 99 | 0 | 2832 | 50 | 9 | 3 | 35 | 1.54 |
| FK-DG2 | *D. gustavii* | 16643-16677 | 18 | 2 | 17 | 94 | 5 | 61 | 48 | 0 | 0 | 51 | 1 |
| FK-DG2 | *D. gustavii* | 16648-16727 | 24 | 3.3 | 25 | 83 | 11 | 96 | 48 | 0 | 0 | 51 | 1 |
| FK-DG2 | *D. gustavii* | 16672-16763 | 21 | 4.2 | 21 | 72 | 13 | 62 | 50 | 0 | 2 | 47 | 1.13 |
| FK-DG2 | *D. gustavii* | 16961-17005 | 22 | 2.1 | 22 | 84 | 8 | 58 | 48 | 0 | 2 | 48 | 1.13 |
| FK-DG9 | *D. gustavii* | 15517-15551 | 18 | 2 | 17 | 94 | 5 | 61 | 48 | 0 | 0 | 51 | 1 |
| FK-DG9 | *D. gustavii* | 15522-15606 | 24 | 3.5 | 25 | **81** | 13 | 99 | 49 | 0 | 0 | 50 | 1 |
| FK-DG9 | *D. gustavii* | 15526-15678 | 48 | 3.2 | 48 | 69 | 23 | 117 | 47 | 0 | 1 | 50 | 1.09 |
| FK-DG9 | *D. gustavii* | 15546-15637 | 21 | 4.2 | 21 | 73 | 13 | 71 | 50 | 0 | 1 | 48 | 1.08 |
| FK-DG9 | *D. gustavii* | 15835-15879 | 22 | 2.1 | 22 | 84 | 8 | 58 | 48 | 0 | 2 | 48 | 1.13 |
| FK-DG9 | *D. gustavii* | 15939-15988 | 23 | 2.1 | 25 | 88 | 7 | 77 | 50 | 0 | 0 | 50 | 1 |
| LK-DG6 | *D. gustavii* | 15024-15741 | 376 | 1.9 | 375 | 98 | 1 | 1395 | 50 | 9 | 4 | 36 | 1.55 |
| LK-DG6 | *D. gustavii* | 15024-15776 | 375 | 2 | 373 | 96 | 2 | 1379 | 50 | 9 | 3 | 36 | 1.54 |
| LK-DG6 | *D. gustavii* | 15891-15926 | 18 | 2.1 | 17 | 94 | 5 | 63 | 50 | 0 | 0 | 50 | 1 |
| LK-DG6 | *D. gustavii* | 15897-15981 | 23 | 3.7 | 24 | 77 | 14 | 81 | 49 | 0 | 0 | 50 | 1 |
| LK-DG6 | *D. gustavii* | 15897-15981 | 24 | 3.5 | 25 | 81 | 13 | 99 | 49 | 0 | 0 | 50 | 1 |
| LK-DG6 | *D. gustavii* | 15891-15981 | 47 | 2 | 46 | 81 | 14 | 109 | 51 | 0 | 0 | 48 | 1 |
| LK-DG6 | *D. gustavii* | 15921-15981 | 22 | 2.8 | 21 | 79 | 13 | 70 | 50 | 0 | 0 | 49 | 1 |
| LK-DG6 | *D. gustavii* | 15967-16012 | 21 | 2.3 | 20 | 85 | 10 | 60 | 47 | 0 | 4 | 47 | 1.21 |
| LK-DG6 | *D. gustavii* | 16210-16254 | 22 | 2.1 | 22 | 84 | 8 | 58 | 48 | 0 | 2 | 48 | 1.13 |
| LK-DG10 | *D. gustavii* | 15514-15549 | 18 | 2.1 | 17 | 94 | 5 | 63 | 50 | 0 | 0 | 50 | 1 |
| LK-DG10 | *D. gustavii* | 15520-15604 | 23 | 3.7 | 24 | 77 | 14 | 81 | 49 | 0 | 0 | 50 | 1 |
| LK-DG10 | *D. gustavii* | 15520-15604 | 24 | 3.5 | 25 | 81 | 13 | 99 | 49 | 0 | 0 | 50 | 1 |
| LK-DG10 | *D. gustavii* | 15514-15604 | 47 | 2 | 46 | 81 | 14 | 109 | 51 | 0 | 0 | 48 | 1 |
| LK-DG10 | *D. gustavii* | 15544-15604 | 22 | 2.8 | 21 | 79 | 13 | 70 | 50 | 0 | 0 | 49 | 1 |
| LK-DG10 | *D. gustavii* | 15590-15635 | 21 | 2.3 | 20 | 85 | 10 | 60 | 47 | 0 | 4 | 47 | 1.21 |
| LK-DG10 | *D. gustavii* | 15833-15877 | 22 | 2.1 | 22 | 84 | 8 | 58 | 48 | 0 | 2 | 48 | 1.13 |
| FK-BM12 | *B. minimum* | 15054-15165 | 40 | 2.9 | 38 | 77 | 9 | 102 | 58 | 0 | 0 | 41 | 1.04 |
| FK-BM12 | *B. minimum* | 15648-15717 | 20 | 3.5 | 20 | 70 | 18 | 56 | 38 | 0 | 0 | 61 | 0.96 |
| FK-BM12 | *B. minimum* | 15731-15772 | 19 | 2.3 | 19 | 88 | 12 | 61 | 54 | 2 | 0 | 42 | 1.13 |
| FK-BM12 | *B. minimum* | 15826-15962 | 72 | 1.9 | 75 | 83 | 7 | 183 | 52 | 2 | 0 | 45 | 1.13 |
| FK-BM12 | *B. minimum* | 15887-15947 | 26 | 2.2 | 29 | 80 | 16 | 76 | 55 | 1 | 0 | 42 | 1.09 |
| FK-BM12 | *B. minimum* | 16068-16119 | 2 | 27.5 | 2 | 77 | 11 | 56 | 46 | 0 | 1 | 51 | 1.12 |
| FK-BM12 | *B. minimum* | 16074-16124 | 18 | 2.8 | 18 | 87 | 3 | 66 | 47 | 0 | 1 | 50 | 1.12 |
| FK-BM12 | *B. minimum* | 16117-16173 | 18 | 3.2 | 18 | 80 | 2 | 71 | 43 | 1 | 3 | 50 | 1.29 |
| FK-BM12 | *B. minimum* | 16353-16503 | 2 | 80.5 | 2 | 68 | 14 | 81 | 49 | 0 | 3 | 45 | 1.25 |
| FK-BM12 | *B. minimum* | 16432-16514 | 24 | 3.6 | 21 | 76 | 15 | 60 | 49 | 0 | 2 | 48 | 1.14 |
| FK-BM15 | *B. minimum* | 15058-15169 | 40 | 2.9 | 38 | 77 | 9 | 102 | 58 | 0 | 0 | 41 | 1.04 |
| FK-BM15 | *B. minimum* | 15652-15721 | 20 | 3.5 | 20 | 70 | 18 | 56 | 38 | 0 | 0 | 61 | 0.96 |
| FK-BM15 | *B. minimum* | 15735-15776 | 19 | 2.3 | 19 | 88 | 12 | 61 | 54 | 2 | 0 | 42 | 1.13 |
| FK-BM15 | *B. minimum* | 15830-15966 | 72 | 1.9 | 75 | 83 | 7 | 183 | 52 | 2 | 0 | 45 | 1.13 |
| FK-BM15 | *B. minimum* | 15891-15951 | 26 | 2.2 | 29 | 80 | 16 | 76 | 55 | 1 | 0 | 42 | 1.09 |
| FK-BM15 | *B. minimum* | 16072-16123 | 2 | 27.5 | 2 | 77 | 11 | 56 | 46 | 0 | 1 | 51 | 1.12 |
| FK-BM15 | *B. minimum* | 16078-16128 | 18 | 2.8 | 18 | 87 | 3 | 66 | 47 | 0 | 1 | 50 | 1.12 |
| FK-BM15 | *B. minimum* | 16121-16177 | 18 | 3.2 | 18 | 80 | 2 | 71 | 43 | 1 | 3 | 50 | 1.29 |
| FK-BM15 | *B. minimum* | 16357-16507 | 2 | 80.5 | 2 | 68 | 14 | 81 | 49 | 0 | 3 | 45 | 1.25 |
| FK-BM15 | *B. minimum* | 16436-16518 | 24 | 3.6 | 21 | 76 | 15 | 60 | 49 | 0 | 2 | 48 | 1.14 |
| LK-PC1 | *P. chalceus* | 14882-14951 | 29 | 2.3 | 30 | 76 | 16 | 74 | 68 | 1 | 0 | 30 | 0.98 |
| LK-PC1 | *P. chalceus* | 14888-14953 | 25 | 2.6 | 26 | 73 | 7 | 64 | 66 | 1 | 0 | 31 | 1.01 |
| LK-PC1 | *P. chalceus* | 15043-15088 | 25 | 2 | 24 | 87 | 12 | 69 | 34 | 17 | 4 | 43 | 1.69 |
| LK-PC1 | *P. chalceus* | 15196-15244 | 20 | 2.4 | 21 | 80 | 6 | 57 | 61 | 0 | 2 | 36 | 1.08 |
| LK-PC1 | *P. chalceus* | 15572-15633 | 14 | 4.5 | 14 | 75 | 15 | 58 | 54 | 3 | 0 | 41 | 1.16 |
| LK-PC1 | *P. chalceus* | 15763-15830 | 29 | 2.6 | 25 | 74 | 24 | 61 | 67 | 1 | 0 | 30 | 0.99 |
| LK-PC1 | *P. chalceus* | 15920-15965 | 25 | 2 | 24 | 87 | 12 | 69 | 34 | 17 | 4 | 43 | 1.69 |
| LK-PC1 | *P. chalceus* | 16073-16121 | 20 | 2.4 | 21 | 83 | 6 | 66 | 63 | 0 | 0 | 36 | 0.95 |
| LK-PC1 | *P. chalceus* | 16084-16121 | 11 | 3.2 | 12 | 82 | 17 | 53 | 65 | 0 | 0 | 34 | 0.93 |
| LK-PC1 | *P. chalceus* | 16262-16352 | 10 | 9.7 | 9 | 74 | 17 | 62 | 47 | 2 | 0 | 50 | 1.13 |
| LK-PC1 | *P. chalceus* | 16329-16376 | 19 | 2.5 | 19 | 80 | 12 | 53 | 54 | 0 | 0 | 45 | 0.99 |
| LK-PC1 | *P. chalceus* | 16346-16401 | 22 | 2.5 | 22 | 78 | 18 | 62 | 55 | 0 | 0 | 44 | 0.99 |
| LK-PC1 | *P. chalceus* | 16388-16432 | 20 | 2.4 | 18 | 92 | 7 | 72 | 53 | 0 | 2 | 44 | 1.13 |
| LK-PC1 | *P. chalceus* | 16430-16464 | 14 | 2.6 | 13 | 86 | 8 | 52 | 37 | 2 | 0 | 60 | 1.12 |
| LK-PC1 | *P. chalceus* | 16473-16511 | 17 | 2.3 | 17 | 86 | 0 | 51 | 46 | 5 | 5 | 43 | 1.48 |
| LK-PC2 | *P. chalceus* | 14882-14951 | 29 | 2.3 | 30 | 76 | 16 | 74 | 68 | 1 | 0 | 30 | 0.98 |
| LK-PC2 | *P. chalceus* | 14888-14953 | 25 | 2.6 | 26 | 73 | 7 | 64 | 66 | 1 | 0 | 31 | 1.01 |
| LK-PC2 | *P. chalceus* | 15043-15088 | 25 | 2 | 24 | 87 | 12 | 69 | 34 | 17 | 4 | 43 | 1.69 |
| LK-PC2 | *P. chalceus* | 15196-15244 | 20 | 2.4 | 21 | 80 | 6 | 57 | 61 | 0 | 2 | 36 | 1.08 |
| LK-PC2 | *P. chalceus* | 15572-15633 | 14 | 4.5 | 14 | 75 | 15 | 58 | 54 | 3 | 0 | 41 | 1.16 |
| LK-PC2 | *P. chalceus* | 15763-15830 | 29 | 2.6 | 25 | 74 | 24 | 61 | 67 | 1 | 0 | 30 | 0.99 |
| LK-PC2 | *P. chalceus* | 15920-15965 | 25 | 2 | 24 | 87 | 12 | 69 | 34 | 17 | 4 | 43 | 1.69 |
| LK-PC2 | *P. chalceus* | 16073-16121 | 20 | 2.4 | 21 | 83 | 6 | 66 | 63 | 0 | 0 | 36 | 0.95 |
| LK-PC2 | *P. chalceus* | 16084-16121 | 11 | 3.2 | 12 | 82 | 17 | 53 | 65 | 0 | 0 | 34 | 0.93 |
| LK-PC2 | *P. chalceus* | 16262-16352 | 10 | 9.7 | 9 | 74 | 17 | 62 | 47 | 2 | 0 | 50 | 1.13 |
| LK-PC2 | *P. chalceus* | 16329-16376 | 19 | 2.5 | 19 | 80 | 12 | 53 | 54 | 0 | 0 | 45 | 0.99 |
| LK-PC2 | *P. chalceus* | 16346-16401 | 22 | 2.5 | 22 | 78 | 18 | 62 | 55 | 0 | 0 | 44 | 0.99 |
| LK-PC2 | *P. chalceus* | 16388-16432 | 20 | 2.4 | 18 | 92 | 7 | 72 | 53 | 0 | 2 | 44 | 1.13 |
| LK-PC2 | *P. chalceus* | 16430-16464 | 14 | 2.6 | 13 | 86 | 8 | 52 | 37 | 2 | 0 | 60 | 1.12 |
| LK-PC2 | *P. chalceus* | 16473-16511 | 17 | 2.3 | 17 | 86 | 0 | 51 | 46 | 5 | 5 | 43 | 1.48 |
| NS-PC1 | *P. chalceus* | 14888-14955 | 29 | 2.6 | 25 | 74 | 24 | 61 | 67 | 1 | 0 | 30 | 0.99 |
| NS-PC1 | *P. chalceus* | 15045-15090 | 25 | 2 | 24 | 87 | 12 | 69 | 34 | 17 | 4 | 43 | 1.69 |
| NS-PC1 | *P. chalceus* | 15198-15246 | 20 | 2.4 | 21 | 80 | 6 | 57 | 61 | 0 | 2 | 36 | 1.08 |
| NS-PC1 | *P. chalceus* | 15387-15477 | 10 | 9.7 | 9 | 73 | 17 | 53 | 47 | 3 | 0 | 49 | 1.18 |
| NS-PC1 | *P. chalceus* | 15471-15526 | 22 | 2.5 | 22 | 78 | 18 | 62 | 55 | 0 | 0 | 44 | 0.99 |
| NS-PC1 | *P. chalceus* | 15513-15557 | 20 | 2.4 | 18 | 92 | 7 | 72 | 53 | 0 | 2 | 44 | 1.13 |
| NS-PC1 | *P. chalceus* | 15555-15589 | 14 | 2.6 | 13 | 86 | 8 | 52 | 37 | 2 | 0 | 60 | 1.12 |
| NS-PC1 | *P. chalceus* | 15598-15636 | 17 | 2.3 | 17 | 86 | 0 | 51 | 46 | 5 | 5 | 43 | 1.48 |
